# The Precautionary Principle for Shift-Work Research and Decision-Making

**DOI:** 10.1093/phe/phy005

**Published:** 2018-03-12

**Authors:** Charleen D Adams, Erika Blacksher, Wylie Burke

**Affiliations:** 1 MRC Integrative Epidemiology Unit (IEU) & School of Social and Community Medicine, University of Bristol; 2 Department of Bioethics and Humanities, University of Washington

## Abstract

Shift work (working outside of 6:00 AM to 6:00 PM) is a fixture of our 24-hour economy, with approximately 18 per cent of workers in the USA engaging in shift work, many overnight. Since shift work has been linked to an increased risk for an array of serious maladies, including cardiometabolic disorders and cancer, and is done disproportionately by the poor and by minorities, shift work is a highly prevalent economic and occupational health disparity. Here we draw primarily on the state of science around shift work and breast cancer to argue that shift work represents a public health threat serious enough to warrant a precautionary stance. We use the precautionary principle to advance our case and view it as a moral compass for shift work research, empowering public health to cast shift work within the domain of health disparities deserving action despite scientific uncertainty. With the precautionary principle, we call for a deliberative decision-making process and formation of a broad shift work research collaboration to protect the health of many millions who work at night.

## Introduction

Shift work (working outside of 6:00 AM to 6:00 PM) is a fixture of our 24-hr economy. Government statistics indicate that approximately 18 per cent of workers in the USA engage in shift work, many overnight (McMenamin, 2007). However, this estimate is unlikely to capture shift work occurring as part of unreported employment, which is done disproportionately by the poor, many of whom are minorities (Saenz, 2008) and by the sometimes otherwise disadvantaged (e.g., middle-aged workers having only a high school education, for whom the mortality rate has been increasing in the USA (Sasson, 2016; Case and Deaton, 2017)). Shift work, in this way, represents a highly prevalent economic and occupational disparity.

Evidence is accruing that shift work is associated with a broad array of adverse health effects, including cardiovascular disease, obesity, metabolic syndrome, type 2 diabetes and cancer (Pan *et al.*, 2011; Gu *et al.*, 2015; Lin *et al.*, 2015; Bass and Lazar, 2016). In 2007, an expert panel at the International Agency for Research on Cancer (IARC) classified shift work involving circadian disruption—the failure to coordinate biological rhythms with the daily light–dark cycle—as a *probable* (2A) carcinogen (International Agency for Research on Cancer, 2007). Although scientific uncertainties remain, studies were carried out in the decade since IARC’s review further strengthen the evidence that shift work is carcinogenic. Together with the increased risks for cardiometabolic diseases and mortality observed among shift workers (Pan *et al.*, 2011; Gu *et al.*, 2015; Lin *et al.*, 2015), this evidence makes shift work a pressing public-health concern.

Because breast cancer is the most studied example of the complex health effects of shift work, we draw on the state of science around this issue to argue that shift work represents a public-health threat serious enough to warrant a precautionary stance. We use the precautionary principle to advance our case and interpret its requirement for ‘shifting the burden of proof to the proponents of an activity’ (Kriebel and Tickner, 2001: 1351), one of its more controversial elements, as a requirement to share responsibility for shift work research, decision-making and policy making through a broad coalition of diverse parties with a stake in shift work. We do not argue that shift work should be avoided until proven safe by shift work’s proponents. We acknowledge that such a requirement is infeasible and contrary to the strong societal value placed on the availability of a range of services that require shift work. Rather, we argue that the principle justifies—and in fact requires—research directed at finding out how we can reduce harms and an advisory process to inform policy making as evidence emerges, with both activities taking into consideration the values of the various individuals and groups with stakes in having shift work as a service and occupational option. On our construal, the principle is respectful and protective of the interests of shift work’s proponents.

We sketch in a preliminary way what this shared responsibility might entail and the obstacles it is likely to face in the US context, and we note how this example might be applicable to other jurisdictions, especially other countries with a similar prevalence of shift work. We begin with a brief overview of the precautionary principle and discussion of its utility as an ethical principle in guiding public-health research and action, building on prior work that has elaborated on the principle in this context (Kriebel and Tickner, 2001; Jordan and O’Riordan, 2004; Pearce, 2004). We focus on the principle’s particular utility as a moral compass for population health science as an interdisciplinary field of research that draws on many disciplines to investigate the disproportionate burden of illness and disease in minority and low socioeconomic status (SES) groups. On our interpretation, the precautionary principle empowers public health to act from a stance of social justice, casting shift work within the domain of health disparities that demand social responsibility and collective action in the face of scientific uncertainty.

## The Precautionary Principle and Population Health Science

The precautionary principle calls for proactive measures to avoid serious harms to human health and ecosystems under conditions of scientific uncertainty. The principle represents a fundamental shift in policy making from a stance of reaction to proven hazards to anticipatory action to prevent potentially serious harms (Martuzzi and Tickner, 2004). This approach challenges basic tenets of the prevailing paradigm of risk assessment, including the assumption that products and activities are safe until proven dangerous and the privileging of private profit over public health and social goods (Mayer *et al.*, 2002).

An oft-cited definition comes from the 1998 Wingspread conference, which states that when activities raise the potential for serious harm to human health, precautionary measures should be taken even in the absence of complete scientific knowledge of cause and effect (Science and Environmental Health Network, 1998). Over the years, the principle has been variously defined and applied (Jordan and O’Riordan, 2004). Four common elements of the principle are: (i) taking preventive action in the face of uncertainty; (ii) shifting the burden of proof to the proponents of an activity; (iii) exploring a wide range of alternatives to possibly harmful actions; and (iv) increasing public participation in decision-making (Kriebel *et al.*, 2005).

Our recommendation for a broad-based consortium that undertakes responsibility for research, deliberative public engagement and making policy proposals draws especially on two tenets (ii and iv) of the principle. The requirement to shift the burden of proof to proponents of the activity in question (ii) is perhaps the most contentious aspect of the precautionary principle (Pearce, 2004). Some commentators have interpreted it to mean that an activity’s proponents must establish that an activity is safe, leading to criticisms that it would stifle innovation and waste resources (Pearce, 2004; Kriebel *et al.*, 2005). Others, however, suggest that a shift in burden of proof does not mean that the activity must cease until proven safe, but rather that proponents of the activity commit to a range of responsibilities, such as thoroughly studying and monitoring potential harms; publicly disclosing information about potential harms; and making restorations for damage done (Schettler and Raffensperger, 2004). The call to study potential harms has been further elaborated as a call for a particular approach to research, one that poses ‘broader hypotheses’, expands ‘characterization of uncertainties’ and studies ‘cumulative and interactive effects as well as risks to vulnerable sub-populations, and preventive interventions’ (Tickner *et al.*, 2003).

These elaborations on the type of research that should be done in the name of precaution make it a particularly apt guide for population health studies, including the shift work studies we describe. Population health science, sometimes referred to as social epidemiology or eco-epidemiology, develops frameworks with the theoretical and methodological power to contextualize biology and behavior within broader social systems to explain the disproportionate incidence of injury, illness and disease, such as are seen among minority and low SES groups (Susser and Susser, 1996). This explanatory enterprise often relies on a notion of social causation in which disease outcomes involve complex interactions and accumulations of exposures to repetitive and mundane, yet chronically stressful, circumstances, such as shift work, that produce a general susceptibility to a multiplicity of diseases (Hertzman and Boyce, 2010). Thus, population health science findings may entail a level of complexity and uncertainty that invite scrutiny not leveled at biomedical research in which singular causes are more directly associated with disease outcomes.

That scrutiny is likely to play out in the public square. Unlike biomedical science, in which findings are typically translated into health knowledge or health-care interventions that rely on individual initiative or resources, population health findings often have implications for community or societal-level policies and practices (e.g., clean air regulations or urban planning). As such they are subject to public debate, both about the credibility of supporting evidence and priorities for public resources and collective action. Such debates are ethical and political in nature, involving a plurality of values related to determinations about what constitutes ‘sound science’ and what aspects of human life most warrant protection and promotion at public cost (Stirling and Gee, 2002; Schettler and Raffensperger, 2004).

Given the likelihood of conflict among such values and need to make normative trade-offs, we interpret the precautionary principle’s call for increasing public participation in decision-making (Tenet iv) in terms of public *deliberation* specifically. Public deliberation is an approach to stakeholder engagement that convenes people from diverse backgrounds for in-depth discussion of topics of public concern to provide policy makers with input about what actions ought to be carried out. Deliberative public engagement that meaningfully involves diverse stakeholders, including shift workers and members of the general public, could benefit decision-making in at least two ways. Individuals with varied perspectives and values are likely to see problems and potential solutions overlooked by experts ‘siloed’ within their fields (Kriebel and Tickner, 2001). Shift workers in particular may have insights regarding potential harms and how they might be mitigated, as we describe in more detail below. In addition, empiric studies show that public deliberation can yield discussions that are well informed and well considered, and recommend solutions that are civic-minded and egalitarian (Abelson *et al.*, 2003; Gastil *et al.*, 2010).

We believe our interpretation of the precautionary principle supports social justice action in two ways. First, we prescribe broad-based research that is ongoing and directed at mitigating harmful effects on vulnerable subpopulations. Second, we call for the inclusive deliberative engagement of stakeholders, especially vulnerable subgroups who bear the burden of potential harms, to ensure their values and needs have the opportunity to be voiced and included in development of policy recommendations.

## Shift Work and Scientific Uncertainty

What we know about shift work is that millions of people engage in it, that it is associated observationally with a modest risk for cancer in multiple cancer sites, as well as other health risks, and that the risk of cancer increases with years of shift work. Additionally, those most likely to do shift work are from minority and low SES groups (Saenz, 2008), which are, in general, exposed to more health risks and experience a greater burden of injury, illness and disease (Braveman *et al.*, 2010). Because much of the extant research into shift work’s negative health effects has been concentrated on breast cancer, we utilize breast cancer as the example to illustrate current knowledge and remaining uncertainties about the health effects of shift work.

### Molecular Mechanisms

Shift work is presumed to have deleterious effects on the body’s circadian rhythms. The circadian molecular clock is a transcriptional–translational feedback loop within each nucleus-containing cell in the body, consisting of a set core of transcription factors whose interactions produce a near 24-hr (endogenous) rhythm. The clock self-regulates its own daily rhythm as well as the daily rhythms of the genes it controls, leading to daily outputs in metabolism, hormone production, energy balance and cellular homeostasis—our circadian rhythms. The external light/dark signal permits the clocks in the brain to coordinate their timing with that of the outside world and to use their coordinated timing as a signal for the rest of the body. Thus, a disturbance in the timing of light (such as by light-at-night or traveling across time zones) can interfere with the timing of the clocks in peripheral tissues. This is largely what is meant by the term chronodisruption or circadian disruption. We experience this as jet lag and malaise, but at the molecular level, the clocks in various tissues and the genes controlled by the clocks are no longer synchronized. Some clock-controlled genes are involved in the cell cycle and the DNA damage response (Sancar *et al.*, 2010). The DNA damage response helps maintain cellular and genetic stability and is an important protection against carcinogenesis (Negrini *et al.*, 2010). Thus, changing the timing of light impacts circadian genetics throughout the body, potentially impacting pathways related to cancer. Eighty per cent of the circadian-disrupting animal studies IARC reviewed demonstrated enhanced carcinogenesis—contributing to IARC’s interpretation that shift work is probably carcinogenic (International Agency for Research on Cancer, 2007). But the exact underlying mechanism, though implicating DNA damage, has yet to be elucidated.

### Epidemiologic Studies

The epidemiologic evidence in humans for the carcinogenicity of shift work is suggestive but inconclusive. Six of the eight shift work and breast cancer studies IARC examined in 2007 demonstrated a modest increase in the risk for breast cancer (see Table 1), mostly among long-term shift workers (International Agency for Research on Cancer, 2007). However, the specific aspects of shift work that contributed to cancer could not be determined, in part due to variable definitions of shift work.

**Table 1.** Shift work and breast cancer studies examined by IARC in 2007

Study	Type	Risk estimate^a^ (extreme group vs. referent)	95 per cent CI	Shift work definition
Schernhammer *et al.* (2001)	Prospective cohort	1.36	1.04–1.78	Rotating (≥3 nights/month + days)
Schernhammer *et al.* (2006)	Prospective cohort	1.79	1.06–3.01	Rotating (≥3 nights/month + days)
Tynes *et al.* (1996)	Nested case-control	1.5	1.1–2.0	Work at night with exposure to artificial light
O’Leary *et al.* (2006)	Case-control	1.04	0.79–1.38	Any evening or overnight work
Davis *et al.* (2001)	Case-control	2.3	1.0–5.3	‘Graveyard’ (either permanent or rotating)
Hansen (2001)	Nested case-control	1.5	1.3–1.7	Night work assigned for trades for which >60 per cent of women estimated to work at night
Lie *et al.* (2006)	Case-control	2.21	1.10–4.45	Years of night work imputed based on nursing jobs outside of hospitals
Schwartzbaum *et al.* (2007)	Retrospective cohort	0.94 (SIR^b^)	0.74–1.18	Night work assigned for job titles for which >40 per cent of staff worked at night

Adapted from International Agency for Research on Cancer (2007).

^a^Odds ratios and relative risks.

^b^SIR = standardized incidence ratio.

Since 2007, there have been six meta-analyses of shift work and breast cancer (Ijaz *et al.*, 2013; Jia *et al.*, 2013; Kamdar *et al.*, 2013; Wang *et al.*, 2013; Lin *et al.*, 2015; Travis *et al.*, 2016). Although one meta-analysis (Travis *et al.*, 2016) saw no evidence for an association between breast cancer and shift work, the results have been questioned because of a focus on older women (many years distant from their shift work exposure), the relatively small number of breast cancer cases included and the variable amount of shift work exposure among study participants (Anon, 2016). With the exception of this article, the overall picture is similar to the studies before 2007, with the additional strength that some revealed a dose effect: the risk of cancer increases with years of shift working.

However, the current data are insufficient to assess whether the effect of shift work on cancer varies by factors that could steer prevention guidelines, a gap in the science that constrains experts from suggesting policies to mitigate harms: namely, by individual characteristics, such as chronotype (preference for engaging in activity earlier or later in the day) and sleep quality; by shift system (rotating or permanent), years on a particular non-day shift schedule, and shift intensity (frequency of shift working; days off between shifts); or by social conditions that allow for undisrupted sleep when not working or access to various services, such as gyms and childcare, factors that could affect shift worker’s abilities to cope with the demands of working at night. In addition, most studies have been performed in European populations, limiting generalizability.

## Justification of a Precautionary Stance

We think the evidence justifies a precautionary stance toward shift work on three grounds. First, as already described, about 21 million people (∼18 per cent) engage in shift work (McMenamin, 2007) in the USA alone, and the body of data on shift work’s effects points to serious and sometimes irreversible harms to health. We focus on cancer in this article, but as noted above, shift workers are also at an increased risk for common chronic morbidity, such as cardiovascular events (Vyas *et al.*, 2012) and type 2 diabetes (Pan *et al.*, 2011). Moreover, the acute risks of sleep deprivation put those who work the night shift at an increased risk for accidents: 32–36 per cent of shift workers fall asleep on the job at least once a week; the risk of occupational accidents is 60 per cent higher for shift workers compared to those who work during the day (Rajaratnam *et al.*, 2013). Together, these shift work-related health risks constitute a serious set of maladies to which a large fraction of the work force is exposed.

Second, the impact of shift work is broad in potential burdens and benefits, affecting many types of services and segments of society. Some forms of shift work, representing essential public safety functions in law enforcement, health care and certain public utilities (e.g., air traffic control), cannot be eliminated. Other less essential forms of shift work (such as 24-hr food stores) may be considered so integral to social goals that they might be difficult to eliminate. They might make major contributions to the economy or modern conveniences and thus be highly valued by some segments of the public at large and by some segments of shift workers.

Third, the health risks of shift work fall disproportionately on members of minority and low SES groups, who are exposed to an array of additional health risks and who experience a disproportionate incidence of preventable morbidity and premature death. These groups may be exposed to health risks associated with, for example, resource-poor neighborhoods that have high levels of pollution and toxins and of violence and crime, institutional and interpersonal discrimination and inadequate health care, all of which put them at heightened risks of poor health (Waitzman and Smith, 1998; Marmot, 2005; Braveman *et al.*, 2010). Additionally, these groups may have few employment options beyond shift work due to low educational attainment, language barriers and discriminatory employment practices. These groups may thus be particularly vulnerable to the effects of shift work, raising concerns about social justice. Although public health and precautionary decision-making are often guided by utilitarian aims to maximize the good of the population at large, concerns about a fair distribution of burdens and benefits are also important in precautionary analyses and in public-health ethics (Comba *et al.*, 2004). Taken together, these considerations—the broad impact of potentially serious health harms, the inability to eliminate shift work and the disproportionate impact on socially disadvantaged groups—support a fundamental shift in how to think about responsibility for shift work research and decision-making.

## Shared Responsibility for Research and Decision-Making through Collaboration and Deliberation

**Figure 1.**
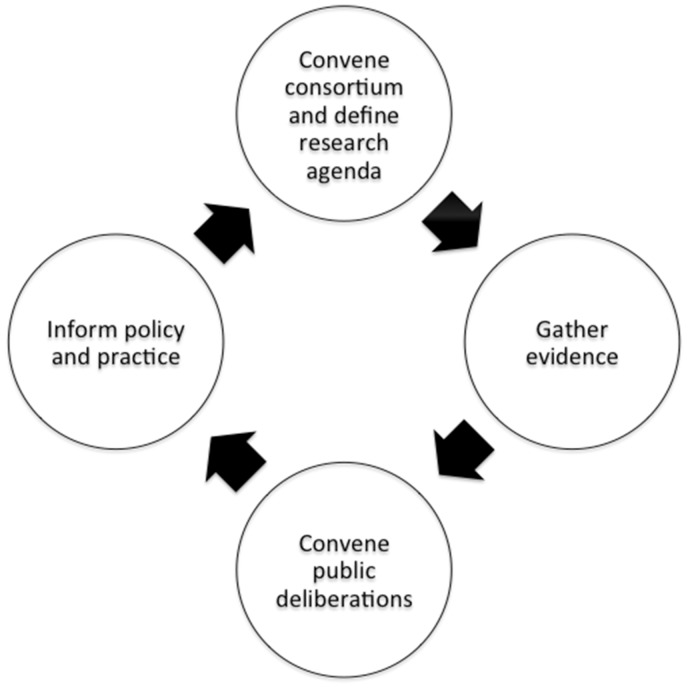
The proposed shift work research consortium and public deliberation.

As described earlier, the precautionary principle’s core tenets include, as two of four key elements, shifting the burden of proof to the proponents of an activity and increasing public participation in decision-making. In the context of the uncertainties and potential trade-offs posed by shift work, we interpret these elements to call for a set of shared responsibilities for ongoing research and a process of deliberative public engagement that informs decision-making and recommends policies, including assurances for those harmed by shift work. We envision a model of shared responsibility for shift work that entails the following phases: convening a research consortium, evaluating current evidence to define critical knowledge gaps, gathering evidence to address those gaps, convening stakeholders for deliberation and using the evidence and deliberative output to inform decision-making about shift work policy and practice (Figure 1). We describe and illustrate each element.

## Research Consortium

We propose the formation of a broad-based consortium and identify key components for a US context—though the model we propose could be adopted by other countries, especially those with a prevalence of shift work similar to that of the USA, and ideally the findings from each country shared in a wider, international setting. But to start, we envision a consortium comprised of diverse US stakeholders to take up a long-term research and action agenda to increase the safety of shift work. This effort would include defining and then investing in research that contributes to knowledge about potential harms and how they might be mitigated, harmonizing definitions of shift work so that data are comparable across settings, publicly disclosing research results and convening various publics to weigh the evidence and deliberate alternatives and options to reduce harms. An ideal public–private convener would be, for example, a partnership between the National Institute for Occupational Safety and Health (NIOSH), the governmental agency whose mission is to develop new knowledge in the field of occupational health and safety and to translate this knowledge for public benefit, and the American Medical Association (AMA), whose members could offer medical expertise sometimes overlooked in population-based approaches to health. NIOSH could then oversee the research process and function as a hub for public deliberation. Other potential public partners include the National Institutes of Health and state and federal public safety and transportation agencies; other potential private partners include industries with a stake in shift work, such as the health-care and transportation industries. The knowledge gained from this process could be used, for instance, in the USA, to inform an Occupational Safety and Health Administration (OSHA) standard for shift work, which currently does not exist.

Our conception of those with a stake in shift work is broad. It includes people who represent public and commercial sectors that employ shift workers, unions, researchers who study the health effects of shift work, governmental agencies that set safety standards for occupations and public health and shift workers themselves. Given that some forms of shift work represent essential public-health safety functions (e.g., law enforcement and air traffic control), are highly valued by the public (e.g., 24-hr access to retail, services and technology), and that its costs may be borne in some ways by the public (e.g., in lost productivity and health-care costs), members of public also have a stake in shift work.

Shift workers should have a special role in the consortium, acting as experts in their own right. There should be ample and regular opportunities for workers to share their insights into the real-world conditions of shift work and to discuss their observations about its effects. Shift workers may be the first to recognize its harmful effects and may have suggestions for mitigating harms that are missed by policy makers and scientists (Stirling and Gee, 2002). In addition, the consortium should foster dialogue among employers and others with the power to effect changes to shift worker schedules, as well as for brainstorming realistic avenues for risk assessment on the job in different types of occupational settings.

Pairing the voices of shift workers and employers with those of scientists, policy makers and others will enable the consortium to develop a research agenda that identifies the types of research projects that are needed to uncover potential alternatives, policies and best practices for shift work. For instance, chronotype is a factor that may impact how workers cope with circadian disruption, and it has been hypothesized that people who work a schedule out-of-synch with their chronotype (for example: people with an evening chronotype who do day shift work and people with a morning chronotype who do night shift work) may be less able to tolerate shift work than those who work in alignment with their chronotype (Erren, 2013). As such, the consortium could make chronotype research an agenda item, though other factors that affect worker’s abilities to adapt to the demands of the night shift or to get quality sleep on days off would likely surface once the members of the consortium start talking to each other. In addition, the consortium might prioritize evaluation of interactions between shift work and other health-related exposures experienced by shift workers.

### Gather Evidence

During this phase, the consortium would plan how do the research identified as central to resolving knowledge gaps needed to inform policy. Broadly, we envision the consortium identifying funding sources (e.g., National Institutes of Health, Centers for Disease Control and Prevention, private foundations); coordinating funding and activities among various research organizations; harmonizing definitions of shift work; setting standards for data acquisition and data sharing; and assuring implementation of the research. As research progresses, it will inevitably produce findings with policy, social and ethical implications that can benefit from a broader deliberative public discussion.

### Convene Stakeholders for Democratic Deliberation

Public deliberation provides the proponents of shift work a voice in the research process and doing so anticipates action on findings, given that shift workers and society would need to be willing to modify their behavior should the research point to serious harms and particular mitigating approaches. To this end, though it is not the only one, formal regulation would be one form of action that we would expect to be explored if findings showed enough harm, and the information and political will gained from public deliberation are likely to increase the probability of regulation passing and to shape the decisions about which types of regulation and which types of less formal policy measures would be suitable in different shift work settings. As such, public deliberation involves representatives from the very groups who have the power to change the culture of shift working and gives them a stake in working with the outcomes of the research. Public deliberation should take place at key moments in research, when evidence seems to warrant action.

Continuing with the example of chronotype, if an increased risk for cancer is verified for night shift workers who have a morning chronotype, the finding would raise difficult questions about what policy or practice to recommend. Approximately 25 per cent of the population is thought to be morning-type, 25 per cent evening-type and the remainder of the population intermediate chronotype (Paine et al., 2006). A recommendation, for example, that morning-types avoid the night shift or that employers avoid hiring morning-types for night shift work may result in a reduced risk of cancer (and other chronic diseases) among these workers, but it would also place *all* the risk on evening- and intermediate-type shift workers. It might also eliminate or greatly reduce employment options for populations that have few employment options and who might value the work despite its health risks. Reducing shift work generally may also harm the economy at large, by reducing efficiencies or the size of the economy. The implications of such a recommendation pose ethical political questions. The effectiveness of any interventions would depend on the nature of the shift work setting, given the complex interactions between shift work and local economies and, more generally, the different values held by members in different shift work settings. For instance, should low-risk populations bear the entire burden of shift work? Should high-risk populations be banned or strongly discouraged from working nightshifts? Should testing for chronotype be offered and, if so, should it be voluntary or mandatory? More broadly, what obligations does an employer have to shift workers to monitor health status or to provide health-care insurance, should they develop cancer or other maladies? The answers to these questions may vary in different shift work settings, as alternatives to shift work, services available to shift workers and costs to employers may vary. By drawing on the values and insights of diverse segments of the public, including those who engage in shift work and those who employ shift workers, shared social values can be identified to inform best shift work practices, and values held more or less strongly in different settings could be identified and harnessed to craft more setting- or region-specific considerations, where appropriate.

### Inform Policy and Practice

After the findings from research on shift work have been publicly deliberated, we envision the consortium formulating and publishing a set of guidelines and recommendations informed by the public values identified in the deliberations. The private–public partnership would disseminate the findings and guidelines broadly throughout the public and private sectors, working closely with state, province and county-level public-health agencies and key figures in the shift work industry (e.g., heads of hospitals and managers) to deliver the message to shift workers and stakeholders within industry.

## Conclusion

We argue that evidence of health harms associated with shift work justifies a precautionary stance. The harms associated with shift work are serious, sometimes irreversible, affect millions of people and fall disproportionately on minorities and the poor, who are exposed to an array of health risks that include but are not limited to shift work. At the same time, shift work enables essential health and public safety functions and offers services of high value to the public. These considerations support a precautionary stance focused on generation of evidence and policies to mitigate harms and identify alternatives.

We have also proposed a model of shared responsibility for shift work research and decision-making undertaken by a consortium of diverse stakeholders to do that work. The idea of shared responsibility for research and action on shift work comports with the very definition of public health—‘what society does collectively to assure the conditions for people to be healthy’ (Institute of Medicine, 2002: xiv). However, while this approach aligns with collectivist values that animate public policy in some countries, it faces considerable obstacles in others, such as the USA, where the political culture prizes the autonomy of individuals and industries over actions that impose limits on these for the good of the population at large. US history is rife with examples of industries—from tobacco and lead paint to asbestos and automakers—actively blocking information about their products’ harms or otherwise shirking responsibility for them (Kurland, 2002). But there have been success stories, and those examples often work through collaborative cross-sectoral models that draw on the expertise, perspectives and commitments of many social sectors and the public at large. One such success story involves bringing together unlikely stakeholders—the National Rifle Association, the Second Amendment Foundation and activists interested in gun suicide, injury prevention and mental health. Their dialogue led to new law in Washington state to develop suicide prevention messaging and training for gun businesses and pharmacies (Stuber, 2016). We believe that protecting public health demands building broad-based coalitions such as this—inclusive of stakeholders with diverse interests and values and representing public and private sectors—that are committed to finding common ground. If the current US political situation has taught us anything, it is perhaps that working and talking within silos lead to polarization that, among other harms, poses serious threats to the public’s health.

## Funding

This work was supported by the Fred Hutchinson Cancer Research Center’s National Cancer Institute-funded ‘Cancer Epidemiology and Biostatistics Training Grant’ [grant number 5T32CA009168]; Cancer Research UK (CRUK) [grant number C18281/A19169]; the Medical Research Council Integrative Epidemiology Unit at the University of Bristol; and the University of Bristol [grant number MC_UU_12013/2].
